# A High Spectral Entropy (SE) Memristive Hidden Chaotic System with Multi-Type Quasi-Periodic and its Circuit

**DOI:** 10.3390/e21101026

**Published:** 2019-10-22

**Authors:** Licai Liu, Chuanhong Du, Lixiu Liang, Xiefu Zhang

**Affiliations:** 1 School of Electronic and Information Engineering, Anshun University, Anshun 561000, China; 2 School of Mechanical and Electrical Engineering, Tarim University, Alar 843300, China; lianglixiu@126.com; 3 School of Mathematics and Computer Science, Guizhou Education University, Guiyang 550018, China; zhang.xie.fu@163.com

**Keywords:** memristor model, memristive chaos, hidden attractor, spectral entropy, multi-type quasi-periodic

## Abstract

As a new type of nonlinear electronic component, a memristor can be used in a chaotic system to increase the complexity of the system. In this paper, a flux-controlled memristor is applied to an existing chaotic system, and a novel five-dimensional chaotic system with high complexity and hidden attractors is proposed. Analyzing the nonlinear characteristics of the system, we can find that the system has new chaotic attractors and many novel quasi-periodic limit cycles; the unique attractor structure of the Poincaré map also reflects the complexity and novelty of the hidden attractor for the system; the system has a very high complexity when measured through spectral entropy. In addition, under different initial conditions, the system exhibits the coexistence of chaotic attractors with different topologies, quasi-periodic limit cycles, and chaotic attractors. At the same time, an interesting transient chaos phenomenon, one kind of novel quasi-periodic, and weak chaotic hidden attractors are found. Finally, we realize the memristor model circuit and the proposed chaotic system use off-the-shelf electronic components. The experimental results of the circuit are consistent with the numerical simulation, which shows that the system is physically achievable and provides a new option for the application of memristive chaotic systems.

## 1. Introduction

Since the discovery of the first chaotic attractor by meteorological scientist Lorenz in 1963 [1], scholars have continued to research and explore new chaotic systems composed of ordinary differential equations. The most representative ones are three-dimensional continuous chaotic systems represented by autonomous ordinary differential equations, such as the Lü system [2,3], Rössler system [4], Chen system [5], and some other typical chaotic systems [6,7,8,9,10,11]. Various four-dimensional chaotic systems or hyperchaotic systems can be obtained by adding linear or nonlinear state feedback controllers based on three-dimensional chaotic systems [12,13,14]. In addition, various multi-wing or multi-scroll chaotic systems can be obtained by modifying multi-segment linear or nonlinear functions to increase the number of exponential two equilibrium points [15,16,17,18].

Distinguishing from the traditional chaotic system which has one or more unstable saddle focal points, hidden attractors are a new type of attractor that has been proposed in recent years. The traditional chaotic system is defined as a self-excited system, and its attractor is newly defined as a self-excited attractor [19]. The hidden attractor is not excited by the unstable equilibrium point, and its attraction basin does not intersect with any unstable equilibrium point, which is the biggest difference from the self-excited attractor [20]. The existence of hidden attractors is found in some stable equilibrium [21,22,23], numerous equilibrium [24,25] or continuous chaotic or hyperchaotic systems with no equilibrium [26,27]. The research of the hidden attraction system has become a new hotspot in the field of non-linear dynamics. It has been shown that the occurrence of hidden attractors is always connected with multistability [19], which exists in many practical engineering systems and may have adverse effects on the system [28,29,30]. It is the first step to uncover all coexisting hidden attractors and then apply an appropriate controlling scheme to keep the system on the desired attractor [19]. Studying the existence of coexisting hidden attractors is very important in engineering applications.

The memristor postulated by L.O. Chua in 1971 is the fourth passive circuit element in electronic circuit theory [31]. The memristor is a two-terminal element, which is divided into magnetic flux and electric charge [32]. Many scholars have shown great enthusiasm for research on the performance of memristors; the memristor theory and its application research has become one of the most popular research topics today. At present, a solid state implementation of memristor has been successfully fabricated in Hewlett-Packard (HP) Labs [33]. However, the manufacturing technology is extremely difficult to implement, resulting in a high cost of memristor, and it cannot be used for commercial purposes. There is a good expectation that memristors will find use in a wide range of applications. In addition, it is necessary to use off-the-shelf components, such as resistor (R), capacitor (C), inductor (L), operational amplifier, analog multiplier, and other components to design a variety of equivalent realization circuits for the memristor [34,35] until the memristor can be manufactured at a low cost. Itoh and Chua replaced the Chua’s oscillator with a piece-wise linear function memristor, standardized the diode in the Chua’s oscillator with a memristor [36], and thus obtained two types of memristor-based chaotic oscillation circuits for the first time. Some scholars have proposed a memristor that possesses a cubic nonlinear [37], a quadratic nonlinear characteristic, and some other improved models [16]. If these models are introduced into a specific circuit, the behavior of the circuit exhibits extremely complex nonlinear behavior, and even chaotic oscillations may occur [37,38,39,40]. The concept of memristors greatly enriches the circuit theory, causing the circuit to have more nonlinear dynamical behavior [32]. As a kind of nonlinear device, memristor has very complex nonlinear behaviours. If it is used to couple with the existing chaotic system, it will be more likely to produce chaotic behavior. It seems that memristive chaotic systems are more suitable for applications in chaotic encryption and other technologies [41], however, there is only a few literature on hidden chaotic systems implemented with memristor models [41,42]. 

Motivated by the research above, a new memristor-based chaotic system and its implementation circuit are constructed in this work. A non-ideal flux-controlled, absolutely non-linear active memristor model is introduced into an existing chaotic system [20]. The proposed system has the phenomenon of a multi-type quasi-periodic limit cycle and multi-attractor chaotic attractors with various topologies, which indicates that the system has numerous hidden attractors and the system is multi-stable. The value of the system spectral entropy (SE) analysis with the parameter changes is about 0.87, which fully outlines the high complexity of this memristive chaotic system. Since the equivalent realization circuit for memristor and the memristive system both are implemented with some off-the-shelf components, it is expected that the system will contribute greatly to further research and applications.

The rest of this paper is organized as follows. Section 2 describes the memristive chaotic mathematical model and analyzes the nonlinear characteristics of the system in terms of phase diagram, time domain diagram, power spectral density, Poincaré map, Lyapunov exponents (LEs), bifurcation diagram, chaotic characteristic graph, and fractal dimension. The bifurcation diagram and the LEs illustrate the extreme multi-stability associated with the initial conditions of the system. The phase diagram reveals the behavior of the infinite number of attractors coexisting with the system. Section 2 also reveals there is a long-term transient anomalous transition behavior for stable chaos under certain initial conditions. The information SE analysis of the memristive chaotic system is given in detail in Section 3. In Section 4, an implementation circuit is designed using common circuit components. It is verified that there are infinite attractors and transient periodic dynamics behavior in memristor chaotic systems, depending on the initial conditions. The conclusion is summarized in Section 5.

## 2. A New 5-D Memristive Chaotic System

### 2.1. Description of the New Memristive Chaotic System

**Figure 1:**
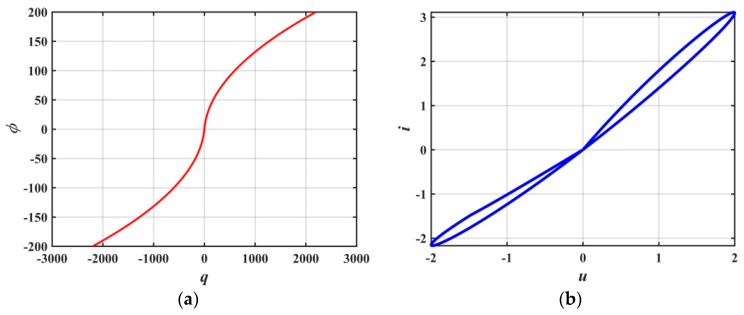
Memristor model: (**a**) the relationship of magnetic flux and charge; (**b**) the I-V characteristic curve.

In this paper, a non-ideal flux-controlled memory proposed in [43,44] is adopted. The mathematical model of the memristor is

(1) $$\left\{ \begin{array}{l} i=W(\phi )u=\left(c+d\left|\phi \right|\right)u\\ \frac{d\phi }{dt}=u-\phi \end{array}\right.$$

where c and d are two positive constant parameters of the memristor, ϕ is the internal state variable of the flux-controlled memristor, W(ϕ) is the conductivity of the memristor, u is the input voltage, and i is the output voltage of the memristor, respectively. According to the flux-controlled theory, when q(ϕ) is the amount of charge, Equation (2) is true. When c=1, d=0.1, and u is a sinusoidal wave voltage with a frequency of 0.3334 Hz, Figure 1a shows the relationship between the magnetic flux and charge of memristor, and Figure 1b shows the pinched hysteresis loop of voltage-current characteristics. Specifically, it can be seen from Figure 1a that the curve passes through the origin in the plane of ϕ−q and monotonically increases; the shape of the I-V characteristic curve is a typical pinched hysteresis loop characteristic with the italics “8” as shown in Figure 1b.

(2) $$\left\{ \begin{array}{l} W(\phi )=c+d\left|\phi \right|\\ q(\phi )=\int_{-\infty }^{\phi }W\left(t\right)dt \end{array}\right.$$

By introducing the memristor model of Equation (1) into the chaotic system shown in [20], a new nonlinear system composed of a memristive model is constructed. The mathematical model of the memristive system is

(3) $$\left\{ \begin{array}{l} \frac{dx}{dt}=ay+xz\\ \frac{dy}{dt}=-bx+yz\\ \frac{dz}{dt}=1-x^{2}-y^{2}\\ \frac{dw}{dt}=-gW(u)w+zw+e\\ \frac{du}{dt}=w-u \end{array}\right.$$

where x, y, and z are the state variables of the system; w is the input voltage of the memristor; u is the internal state variable of the memristor; and a, b, c, d, e, g are positive real parameters of the system. The equilibrium points of system (3) can be solved by Equation (4).

(4) $$\left\{ \begin{array}{l} ay+xz=0\\ -bx+yz=0\\ 1-x^{2}-y^{2}=0\\ -gW(u)w+zw+e=0\\ w-u=0 \end{array}\right.$$

Equation (4) can be simplified and reorganized as

(5) $$\left\{ \begin{array}{l} ay^{2}+bx^{2}=0\\ y^{2}+x^{2}=1 \end{array}\right.$$

Equation (5) has no solution since a and b are positive real parameters. Lyapunov exponents (Les) is an effective means to quantitatively study chaotic systems. Its positive and negative values in a certain direction indicate the degree of the average divergence or convergence within the adjacent orbits of the attractor over a period of time. In order to solve the Les of the system (3) using Wolf algorithm [45], the Jacobian matrix of system (3) is obtained as

(6) $$J=\left[\begin{array}{ccccc} z & a & x & 0 & 0\\ -b & z & y & 0 & 0\\ -2x & -2y & 0 & 0 & 0\\ 0 & 0 & w & z-g\left(c+d\left|u\right|\right) & -gdw\cdot sign\left(u\right)\\ 0 & 0 & 0 & 1 & -1 \end{array}\right]$$

The system parameters are chosen as a=1, b=0.05, c=1, d=0.1, e=1, and g=1, and the initial value is set as $\left(x_{0},y_{0},z_{0},w_{0},u_{0}\right)=\left(-1,-1,0,0,1\right)$. Using the ode45 numerical solver, the Les of the proposed chaotic system are $LE_{1}=0.034291$, $LE_{2}=-0.036091$, $LE_{3}=0$, $LE_{4}=-0.93593$, and $LE_{5}=-1.2806$, respectively. Except for one positive and one zero Lyapunov exponent, the other Les are negative, and the sum of all Lyapunov exponents is −2.21833. The Les show that the whole phase volume of the system is exponentially shrinking rapidly, so the system is chaotic under the above parameters. At this point, the Kaplan-Yorke dimension of the system is shown in Equation (7).

(7) $$D_{KY}=4+\frac{LE_{1}+LE_{2}+LE_{3}+LE_{4}}{\left|LE_{5}\right|}=3.2677$$

**Figure 2:**
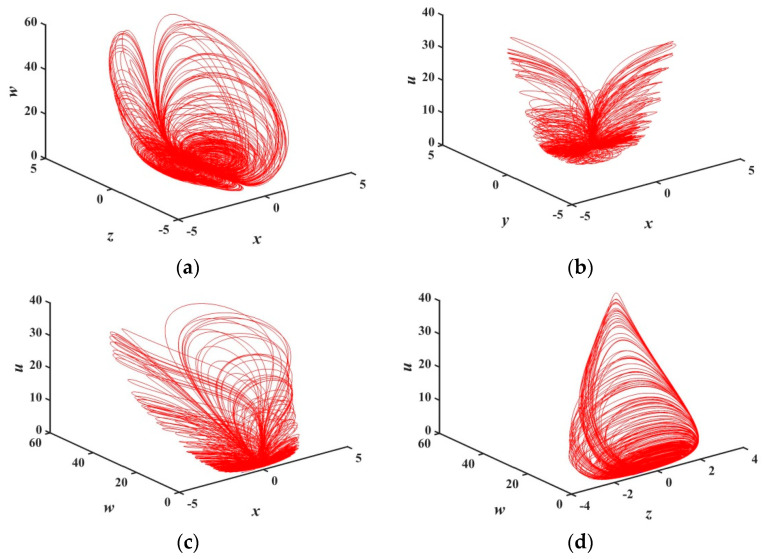
3-D chaotic attractor of system (2): (**a**) x−z−w space, (**b**) x−y−u space, (**c**) x−w−u space, and (**d**) z−w−u space.

**Figure 3:**
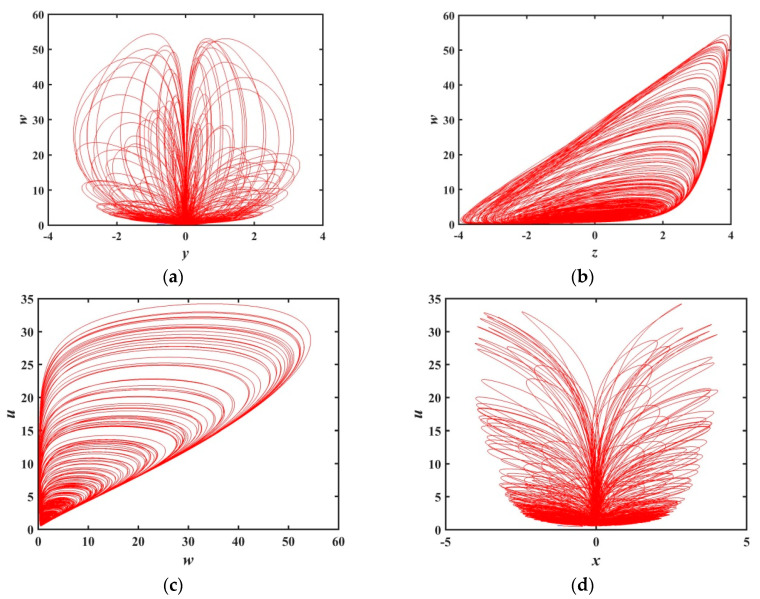

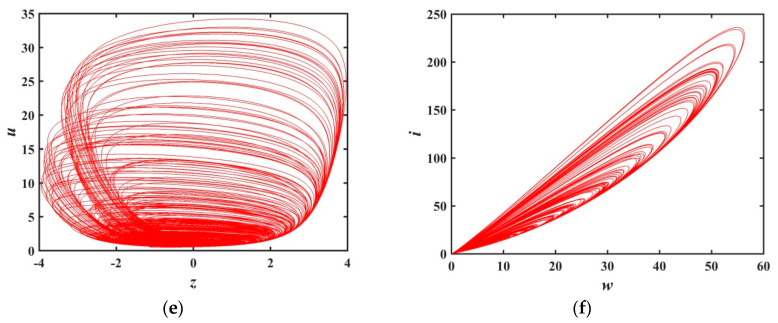
2-D chaotic attractor of system: (**a**) y−w plane; (**b**) z−w plane; (**c**) w−u plane; (**d**) x−u plane; (**e**) z−u plane; and (**f**) w−i plane of memristor model (1).

Under the above parameters, the step size h=0.001 and simulation time t=2000 seconds, the three-dimensional attractor phase diagrams and the two-dimensional attractor phase diagrams of the system are shown in Figure 2 and Figure 3, respectively. Among them, Figure 3f is the chaotic attractor on the terminal voltage w and the current flowing i of the memristor on the plane of w−i for the chaotic system, reflecting the nonlinear dynamic characteristics of the memristor element. It can be seen from the attractor phase diagrams that the system has new hidden attractors which are different from other chaotic systems. Since such attractors have not been reported in previous literature, the proposed system enriches the types of hidden chaotic attractors and provides a broader option for the application of memristor-based chaotic systems.

**Figure 4:**
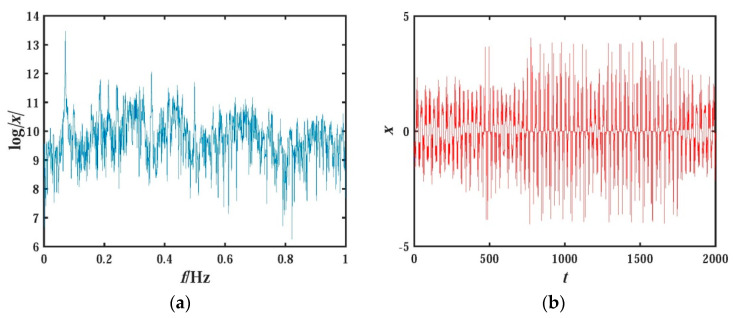
Frequency spectrum and time series of x variable for system (2): (**a**) frequency spectrum; (**b**) time series.

The two graphs shown in Figure 4 are the power spectral density and time domain diagram of the state variable x for the system (3). The power spectral density curve is continuous and has no sharp peaks; the time series diagrams are aperiodic, which accords with the characteristics of chaos. 

**Figure 5:**
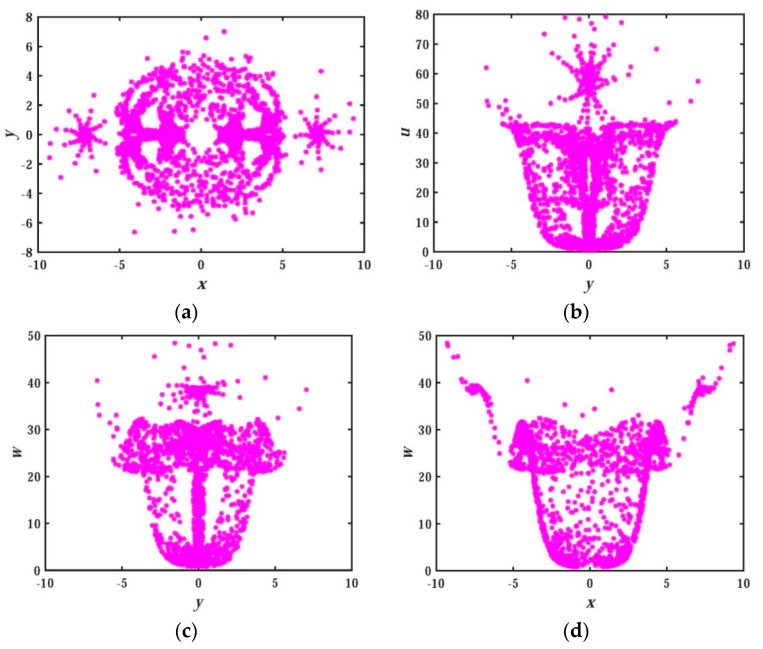
Poincaré map of system (2): (**a**) x−y plane; (**b**) y−u plane; (**c**) y−w plane; (**d**) x−w plane.

Here we continue to analyze the nonlinear behavior of System (3) using Poincaré map [46,47,48]. Figure 5 displays the Poincaré maps on the plane of x−y, y−u, y−w and x−w in z=0 cross section. The shape of the curve is composed of dense point sets, which shows that the system conforms to the characteristics of a chaotic system. In addition, it can be clearly seen that these point sets constitute a variety of graphic shapes, indicating that the attractors have extremely complicated folding behavior in the phase space and the hidden attractor itself has a complex topological structure. The Poincaré maps of the memristor-based hidden chaotic system are much more complex than any other Poincaré maps reported in the previous literature, that is, the Poincaré map with such a complex structure has rarely been reported in the existing literature. If the system is used as a signal generator, it will have a strong application prospect in chaotic encryption, chaotic communication, and other engineering fields. 

### 2.2. Bifurcation Diagram with a as Varying Parameter

**Figure 6:**
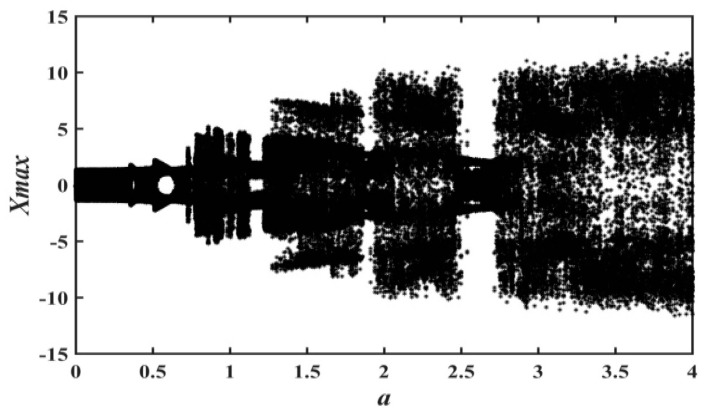
Bifurcation diagram of x versus a for system (2) when b=0.05, d=0.1, c=e=g=1, $\left(x_{0},y_{0},z_{0},w_{0},u_{0})=(-1,-1,0,0,1\right)$, and a∈(0,4).

**Figure 7:**
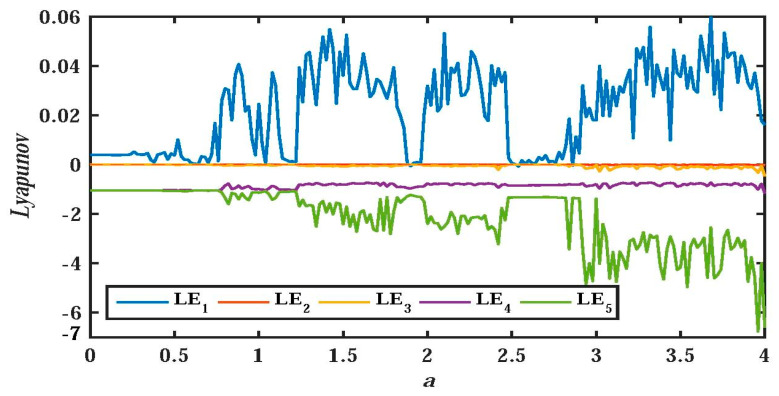
Les of system (2) versus a, when b=0.05, d=0.1, c=e=g=1, $\left(x_{0},y_{0},z_{0},w_{0},u_{0})=(-1,-1,0,0,1\right)$, and a∈(0,4).

In order to uncover the relationship between system parameters and nonlinear behavior of system dynamics, the research method of the bifurcation diagram for the proposed system will be adopted in this section. At the same time, the Les and the attractor’s phase diagram are also used to graphically analyze the nonlinear dynamic behavior of the system. Figure 6 and Figure 7 depict the bifurcation diagram and Les diagram, where fixing the system parameters, b=0.05, d=0.1, c=e=g=1, and the initial value $\left(x_{0},y_{0},z_{0},w_{0},u_{0})=(-1,-1,0,0,1\right)$, changing the value of parameter a, and a∈(0,4). With the increase of system parameter a, Figure 7 shows that the system is in the alternate change from non-chaotic state to chaotic state, and the system is in a large range of a chaotic state.

**Figure 8:**
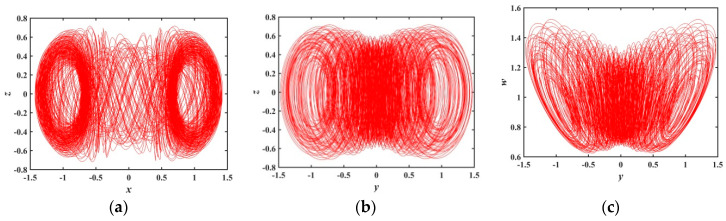
Projections of 2-D phase diagram with parameter a=0.3: (**a**) attractor on the x−z plane; (**b**) attractor on the y−z plane; (**c**) attractor on the y−w plane.

During a∈(0,0.3761), the system has small positive maximum Les with almost no fluctuation, and the system is in a weak chaotic state. The phase diagrams are shown in Figure 8, where a=0.3, $LE_{1}=0.0023524$, $LE_{2}=-0.0028756$, $LE_{3}=0$, $LE_{4}=-1.0479$, and $LE_{5}=-1.0494$.

**Figure 9:**
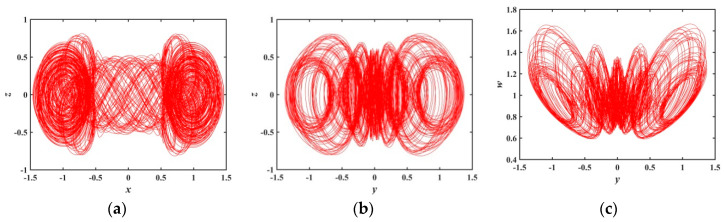
Projections of a 2-D phase diagram with parameter a=0.4: (**a**) attractor on the x−z plane; (**b**) attractor on the y−z plane; (**c**) attractor on the y−w plane.

During a∈(0.3761,0.6301), Figure 6 shows that the largest Les in this range fluctuate within a very small range, indicating that the orbit formed by the trajectory of the system is slowly evolving. Figure 9 represents the phase diagram when a=0.4, which shows how the attractor with higher recognition evolves from the attractor when a=0.3.

**Figure 10:**
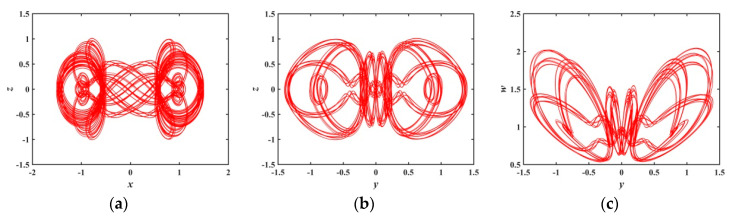
Projections of 2-D phase diagram with parameter a=0.70: (**a**) attractor on the x−z plane; (**b**) attractor on the y−z plane; (**c**) attractor on the y−w plane.

**Figure 11:**
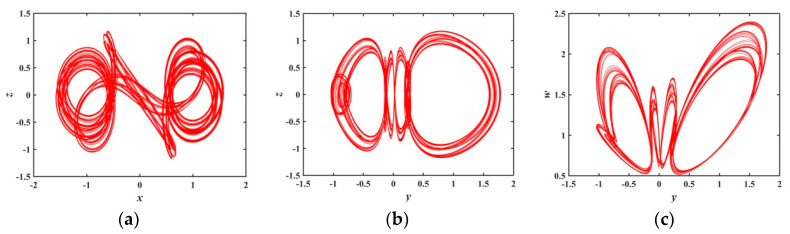
Projections of 2-D phase diagram with parameter a=0.75: (**a**) attractor on the x−z plane; (**b**) attractor on the y−z plane; (**c**) attractor on the y−w plane.

When a∈(0.6301,0.7597), Figure 6 presents that there are several periodic windows in the system, and the Les of the system approach to zero at the periodic window. Figure 10 and Figure 11 show the phase diagrams for a=0.70 and a=0.75, respectively. These phase diagrams show that the phase diagrams corresponding to the two values are all quasi-periodic limit cycle states. Because the shape of these two limit cycles is quite different, it indicates that the system has extremely complex and diverse hidden attractors.

In the range of a∈(0.7597,1.0386), the bifurcation diagram of Figure 6 indicates that the system is in a chaotic state, which has been demonstrated from Figure 2 to Figure 5 when a=1 in the previous discussion. 

**Figure 12:**
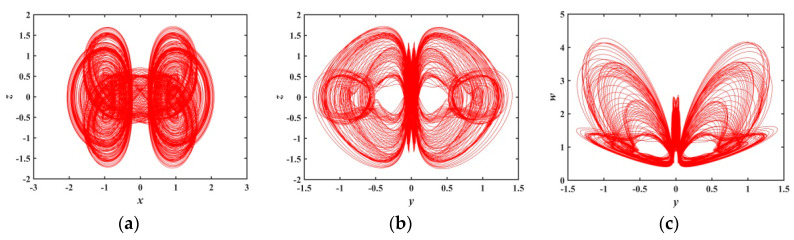
Projections of 2-D phase diagram with parameter a=1.17: (**a**) attractor on the x−z plane; (**b**) attractor on the y−z plane; (**c**) attractor on the y−w plane.

In the range of a∈(1.0386,1.1782), there is a positive LEs in Figure 7, hence, the system behaves as a chaotic state. When a=1.17, we calculate the LEs, $LE_{1}=0.0010416$, $LE_{2}\approx LE_{3}\approx 0$, $LE_{4}=-1.021$, and $LE_{5}=-1.0893$, respectively. Figure 12 is a phase diagram when a=1.17. We are surprised to find a very interesting hidden attractor from the phase diagram, as shown in Figure 12. The shape of the attractor is different from the ones when a=1, and these attractors have never been reported in previous literature, reflecting the novelty and diversity of system attractors. The new attractor further illustrates that the system has very complex nonlinear behavior.

When a∈(1.1782,1.2179), Figure 6 and Figure 7 show the existence of quasi-periodic limit cycles in the system, and the largest Les equals to zero at a=1.2149.

**Figure 13:**
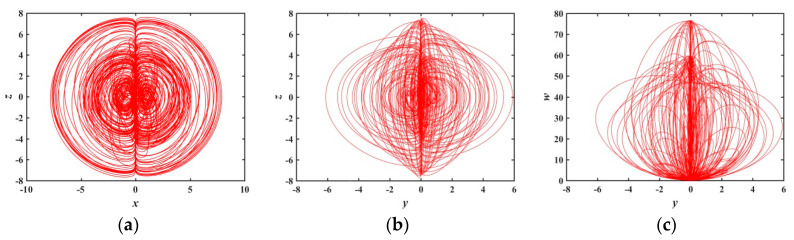
Projections of 2-D phase diagram with parameter a=1.28: (**a**) attractor on the x−z plane; (**b**) attractor on the y−z plane; (**c**) attractor on the y−w plane.

When a∈(1.2179,1.8804), Figure 7 shows that the system has large positive Les, so the system is in a chaotic state. Figure 13 is a phase diagram of the system at a=1.28, in this case, $LE_{1}=0.034289$, $LE_{2}=-0.033743$, $LE_{3}=0$, $LE_{4}=-0.78208$, and $LE_{5}=-1.6299$. The shapes of the hidden attractors shown in the Figure 13 are also different from the ones analyzed earlier, which further confirms the novelty and diversity of the hidden attractors in the memristive system.

**Figure 14:**
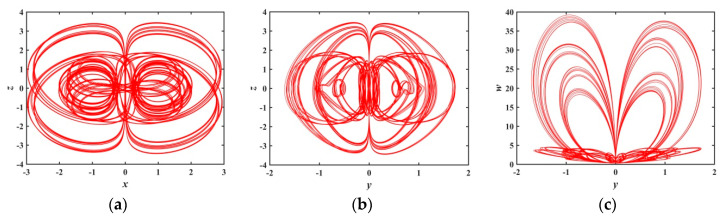
Projections of 2-D phase diagram with parameter a=1.891: (**a**) attractor on the x−z plane; (**b**) attractor on the y−z plane; (**c**) attractor on the y−w plane.

**Figure 15:**
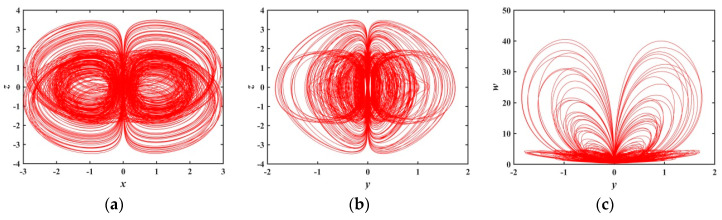
Projections of 2-D phase diagram with parameter a=1.89678: (**a**) attractor on the x−z plane; (**b**) attractor on the y−z plane; (**c**) attractor on the y−w plane.

When a∈(1.8804,1.9651), Figure 6 shows that there is a transition from a non-chaotic state to a chaotic state. At a=1.891 and a=1.89678, the phase diagrams of the system are shown as Figure 14 and Figure 15, respectively, which demonstrates that the system evolves from non-chaotic state to chaotic state. Under this condition, the shapes of the quasi-periodic limit cycle and chaotic attractor exhibited are different from the ones discussed above. Therefore, the system has a myriad of hidden attractors when increasing the value of a. It reflects the strong randomness and high complexity of the system after the introduction of memristors.

**Figure 16:**
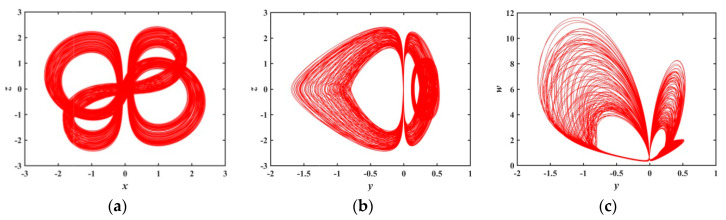
Projections of 2-D phase diagram with parameter a=2.56: (**a**) attractor on the x−z plane; (**b**) attractor on the y−z plane; (**c**) attractor on the y−w plane.

**Figure 17:**
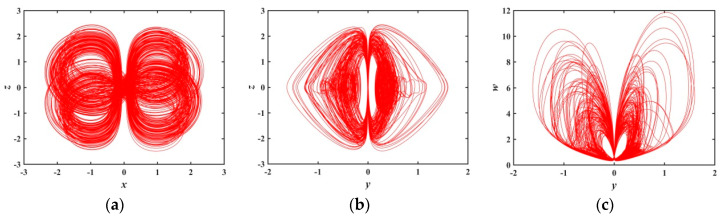
Projections of 2-D phase diagram with parameter a=2.65: (**a**) attractor on the x−z plane; (**b**) attractor on the y−z plane; (**c**) attractor on the y−w plane.

Figure 6 illustrates when a∈(1.9651,2.4981), the system is in a chaotic state. During a∈(2.4981,2.8597), we can see from Figure 6 that the system behaves as a complex bifurcation. During this range, the system has small Les shown as in Figure 7, and the system is in the transition stage from non-chaotic state to a chaotic state. Figure 16 and Figure 17 are the phase diagrams of the system at a=2.56 and a=2.65, respectively. In the meantime, phase diagrams also show the transition from quasi-periodic limit cycle to a chaotic state. The shape of the attractor is different from that of the previous one, which proves that there are numerous hidden attractors and complex topological structures with the change of system parameters. For the range of a∈(2.8597,4), the bifurcation diagram reveals that the system is in a chaotic state.

Based on the above analysis, the shape of the novel quasi-periodic limit cycles and novel attractors of the system have never been reported in existing literature. According to the new chaotic system standard guidelines that “The system should exhibit some behavior previously unobserved” [49], proposed by Sprott, J., C., we could conclude that the system is a new chaotic system. And the occurrence of many quasi-periodic attractors with a single parameter variation is also very rare in other hidden chaotic systems. All these simulations prove that the system has innumerable hidden chaotic attractors and extremely complex attractor topology, which greatly enriches the types of chaotic attractors and provides theoretical support for engineering applications in communication security.

### 2.3. Analysis of Multi-Stability

**Figure 18:**
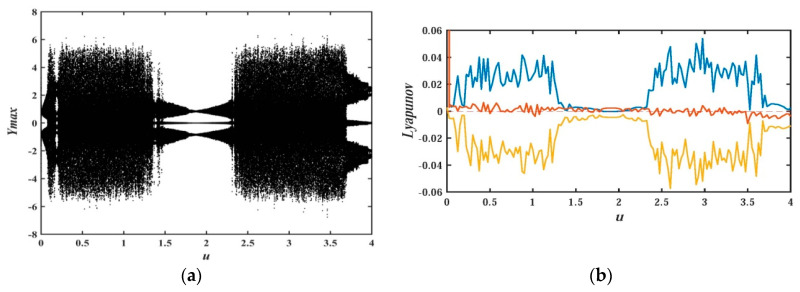
Bifurcation diagram of y and Les versus u for system (2) with initial value $O_{0}=(x_{0},y_{0},z_{0},w_{0},u_{0})=(u,0,0,0,0)$, and u∈[0,4]: (**a**) bifurcation diagram; (**b**) Les graph.

**Figure 19:**
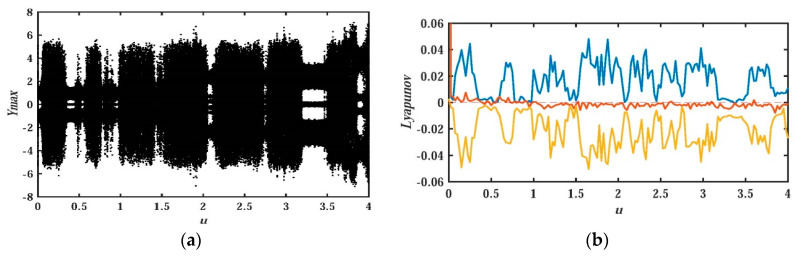
Bifurcation diagram of y and Les versus u for system (2) with initial value $O_{1}=(x_{0},y_{0},z_{0},w_{0},u_{0})=(u,u,0,0,0)$, and u∈[0,4]: (**a**) bifurcation diagram; (**b**) Les graph.

Keeping the system parameters unchanged, it is important to analyze the influence of the initial value of the system on the dynamic behavior of the memristor-based chaotic system with hidden attractors. The system parameters in this section are a=1, b=0.05, d=0.1, *c* = *e* = *g* = 1.The bifurcation diagram and LEs diagram of the system at different initial values $O_{0}=(x_{0},y_{0},z_{0},w_{0},u_{0})=(u,0,0,0,0)$ and initial value $O_{1}=(x_{0},y_{0},z_{0},w_{0},u_{0})=(u,u,0,0,0)$ are shown in Figure 18 and Figure 19, respectively.

For the initial value of $O_{0}=(x_{0},y_{0},z_{0},w_{0},u_{0})=(u,0,0,0,0)$, it is interesting that the bifurcation graphs are axisymmetric about the vertical axis during u∈(−4,0) and u∈(0,4). Readers who are interested could test symmetry by themselves. The bifurcation diagram at u∈(0,4), as shown in Figure 18a and the corresponding Les, is depicted in Figure 18b. In order to reflect the Les more clearly, only the range of LEs∈(−0.06,0.06) is given in Figure 18b and Figure 19b. As for the part of LEs∈(−2.5,−0.06), it is not given. It can be seen from the two bifurcation diagrams that there are different bifurcation shapes and countless attractors for the proposed system corresponding to different initial values.

**Figure 20:**
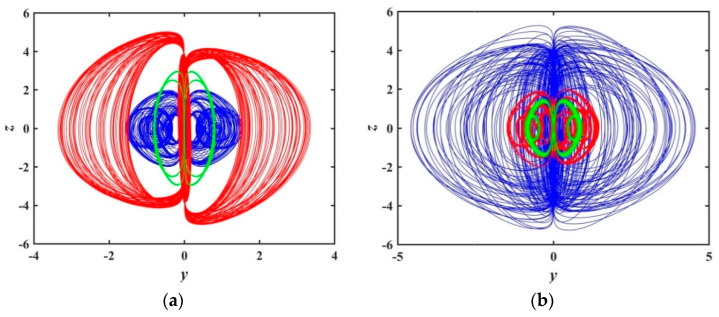
Phase diagram on the y−z plane for system (2) with different initial values: (**a**) (0.51,0.51,0,0,0) (blue), (3.34,3.34,0,0,0) (red), (0.01,0,0,0,0) (green); (**b**) (3,3,0,0,0) (blue), (0.9711,0.9711,0,0,0) (red), (1.922,0,0,0,0) (green).

Figure 20 gives the projection of hidden attractors for the system (3) on the y−z phase plane with different initial values. The blue curve of Figure 20a corresponds to the initial value of (0.51,0.51,0,0,0); the Les are $LE_{1}=0.013492$, $LE_{2}=0$, $LE_{3}=-0.017959$, $LE_{4}=-0.98013$, and $LE_{5}=-1.1965$, respectively. The system is in a chaotic state with hidden attractors; the red curve corresponds to the initial value of (3.34,3.34,0,0,0); the Les of the system are $LE_{1}=-0.0080971$, $LE_{2}=0$, $LE_{3}=-0.0014535$, $LE_{4}=-0.73827$, and $LE_{5}=-2.0881$. The phase diagram shows that the system is in a quasi-periodic state, and the attractor is formed by a high density curve. The green curve corresponds to the initial value of (0.01,0,0,0,0), the system is in the cycle limit cycle state. In Figure 20b, the blue curve corresponds to the initial value of (3,3,0,0,0), the corresponding Les are $LE_{1}=0.032934$, $LE_{2}=0$, $LE_{3}=-0.035035$, $LE_{4}=-0.78493$, and $LE_{5}=-1.7115$, and the system is in the chaotic state; the red curve corresponds to the initial value of (0.9711,0.9711,0,0,0), the corresponding Les are $LE_{1}=-0.0027992$, $LE_{2}=LE_{3}=0$, $LE_{4}=-1.0162$, and $LE_{5}=-1.1027$; the system is in quasi-periodic state. The green curve corresponds to the initial value of (1.922,0,0,0,0); the Les of the system are $LE_{1}=0.0017476$, $LE_{2}=0$, $LE_{3}=-0.0012311$, $LE_{4}=-1.0485$, and $LE_{5}=-1.0662$, the system is in a weak chaotic state. 

Based on the analysis of the two values of $O_{0}$ and $O_{1}$, we find different initial values have a great influence on the dynamic behavior of the system (3). 

**Figure 21:**
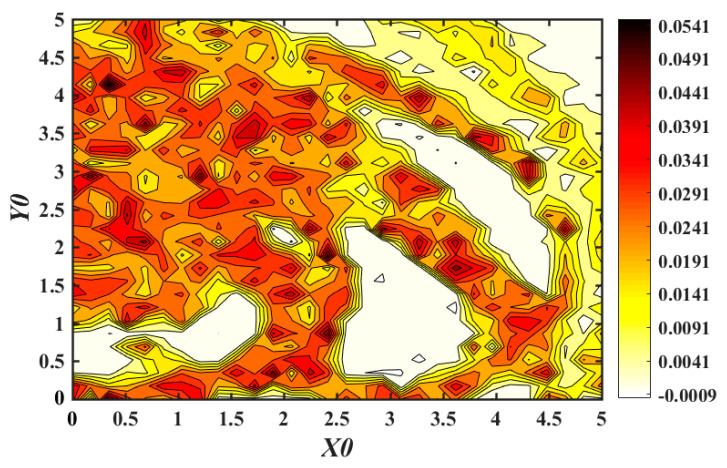
Multi-stable nonlinear dynamic behavior distribution of chaotic for memristive system with different initial values.

In order to reflect more clearly the sensitivity of the memristive system, which is more susceptible to the initial value, Figure 21 shows the multi-stable nonlinear dynamic behavior distribution of chaotic for the system (3) at $x_{0}\in \left(0,5\right)$ and $y_{0}\in \left(0,5\right)$ when the initial value is $\left(x_{0},y_{0},0,0,1\right)$. In the range of maximum Lyapunov index from −0.0009 to 0.0541, the different dynamic behavior of the system corresponds to different colors as marked in Figure 21. There are obvious boundaries between chaotic and non-chaotic regions in the figure. When the system is under different initial conditions, the system exhibits a quasi-periodic state, weak chaotic state, chaotic state and so on. This indicates that there is complex multi-attractor coexistence in the system. 

From the analysis of Figure 20 and Figure 21, we could easily find the coexistence of multiple attractors, which indicates that the system has numerous multiple attractors, and there is multi-stability phenomena. Specifically, figures show the coexistence of chaotic attractors with different topologies and the coexistence of quasi-periodic limit cycles and chaotic attractors. At the same time, there is a quasi-periodic limit cycle and multi-attractor phenomenon of chaotic attractors with various topologies. These simulations and calculations prove that the proposed memristive chaotic system has very rich and complex hidden dynamics. In addition, it should be pointed out that the above analysis is only two numerical examples for different initials, since there are infinite cases of initial value and the dynamic multi-stability characteristics of the system are much more complicated than what is exhibited. Due to the limitation of workload, no more detailed analysis and discussion will be made here, and further research may be conducted later.

### 2.4. Analysis of Transient Chaos

A transient chaotic state is a special kind of chaotic state which refers to the phenomenon that under some certain parameters the system gradually evolves into the periodic state from a chaotic state after a long observation period [50,51,52]. The phenomenon of transient chaos discussed in this section does not occur in the original hidden system [20]. 

**Figure 22:**
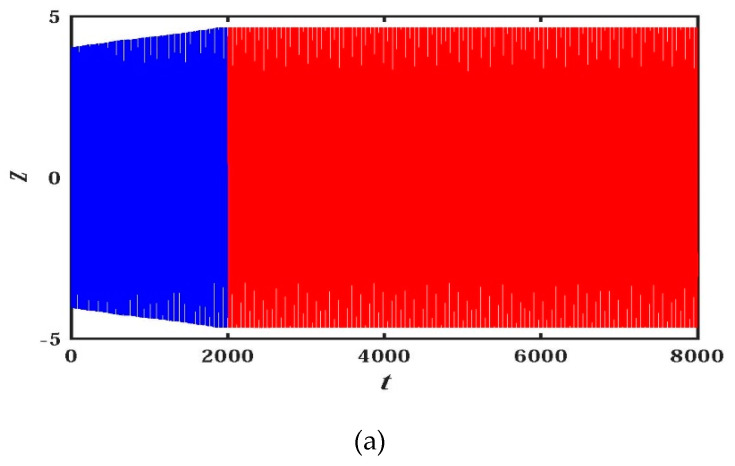

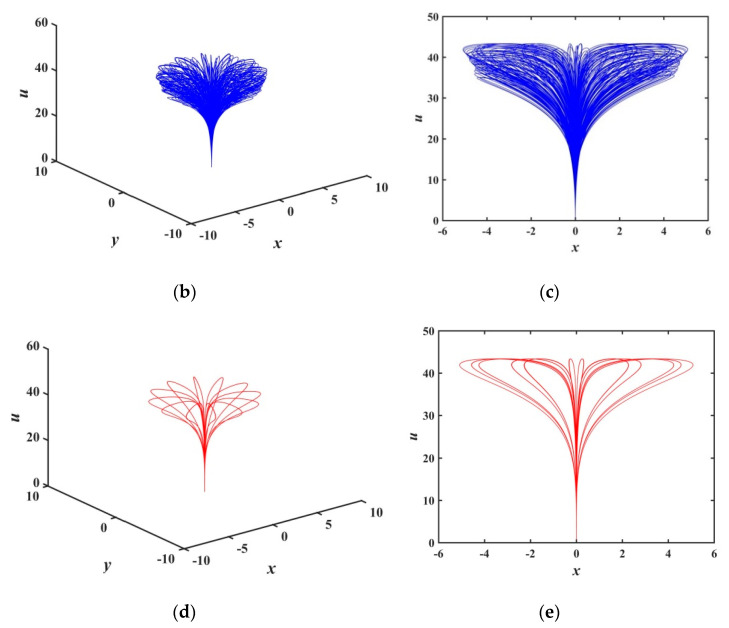
Time series and phase diagram of attractors for transient chaotic: (**a**) time series of z when t∈(0,8000); (**b**) phase diagrams in the x−y−u space when t∈(0,2000); (**c**) phase diagrams on the x−u plane when t∈(0,2000); (**d**) phase diagrams in the x−y−u space when t∈(2000,8000); (**e**) phase diagrams on the x−u plane when t∈(2000,8000).

When the parameters are a=b=0.05, c=e=g=1, and d=0.1, the initial value is $\left(x_{0},y_{0},z_{0},w_{0},u_{0}\right)=\left(-1,-1,4,4,4\right)$; the transient behavior is shown in Figure 22, and the observation time is t=8000 seconds. In Figure 22a, the blue curve color corresponds to t∈(0,2000), and the red curve corresponds to t∈(2000,8000). Under the above initial value and parameters, and during t∈(0,2000), the system LEs are $LE_{1}=0.0043476$, $LE_{2}=0$, $LE_{3}=-0.0015311$, $LE_{4}=-1.0434$, and $LE_{5}=-1.7777$, respectively. The system appears as a weak chaotic state, and the projections of phase diagram for the hidden chaotic attractors in the x−y−u space and on the x−u plane are respectively shown as Figure 22b,c. Compared with Figure 3d, it is found that the hidden attractors are very different at this time, and they belong to novel and interesting attractors. 

During t∈(2000,8000), the periodic phenomena can be seen from the time domain diagram of Figure 22a, and the LEs of the system can be quantitatively calculated to be $LE_{1}=LE_{2}=LE_{3}=0$, $LE_{4}=-1.0451$, and $LE_{5}=-1.7766$, respectively. Therefore, the system is in a quasi-periodic state, and the projections corresponding to the hidden periodic state attractors on the x−y−u space and x−u plane are illustrated in Figure 22d,e. The quasi-periodic attractor is different from the quasi-periodic attractor that appears earlier: The quasi-periodic attractor evolves from chaotic attractor to quasi-periodic attractor in the process of transient chaos. The shape of the attractor is quite different from the quasi-periodic attractor seen before, and it is a more representative, novel and interesting quasi-periodic attractor. The new quasi-periodic attractor is also an unexpected discovery during analyzing transient chaos.

## 3. Entropy Analysis for Memristive Chaotic Systems

In the above analysis, we have learned that the system composed of memristors with hidden attractors has abundant nonlinear dynamic behaviors. In this section, we will quantitatively evaluate the complexity of the system (3) by means of information SE. The detailed SE algorithm is studied in the literature [20,53]. As an excellent algorithm in structural complexity, SE algorithm is a powerful measure of the chaotic characteristics of the system and can better measure the structural complexity of the high-dimensional chaotic system as a whole. Therefore, we adopt the SE algorithm to value the structural complexity of the system in terms of parameters and initial values.

### 3.1. SE Analysis Depending on Parameters 

**Figure 23:**
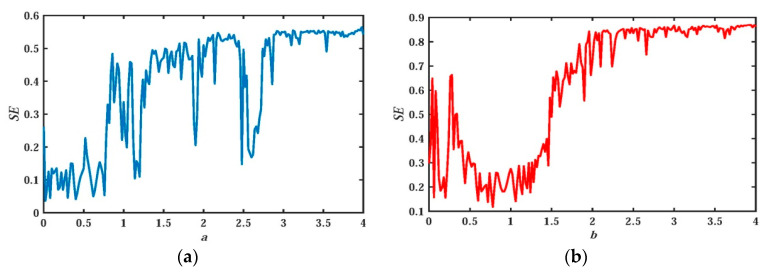
SE with different a and b: (**a**) SE vs. a (b=0.05); (**b**) SE vs. b (a=1) .

In Section 2.2, the abundant nonlinear dynamic characteristics of the system are analyzed in detail with the variation of system parameter. It was proved that the change of system parameter a can greatly affect the dynamic characteristics of the system and then affect the nonlinear complexity of the system. The effect of changes in parameters a and b on SE will be quantitatively analyzed in this section. In order to ensure the consistency of system parameters, system parameters are fixed as c=e=g=1, d=0.1, and initial value $\left(x_{0},y_{0},z_{0},w_{0},u_{0}\right)=\left(-1,-1,0,0,1\right)$. The variation of SE with system parameters a and b is plotted as shown in Figure 23.

When b=0.05 and a∈(0,4), the change of SE is shown in Figure 23a, where there are large fluctuations in the curve, indicating that the change of a has a great impact on structural complexity. The maximum SE of the system fluctuates slightly around 0.55 with the change of a. The amplitude change of the trend for the whole curve is completely consistent with Figure 6 and Figure 7, which further verifies the correctness of the previous analysis.

When a=1 and b∈(0,4), the change of SE is shown in Figure 23b, where few periodic windows exist, reflecting that the system is insensitive to parameter changes. That is to say, with the change of b, the system has a large continuous chaotic interval and a large non-chaotic interval. So it is convenient to obtain the hidden chaotic state of the system, which provides the possibility for engineering application. In addition, the SE value of the system in Figure 23b fluctuates within a very small range around 0.87, which is obviously larger than the SE shown in Figure 23a. In Liu’s original system [20], the value of SE is 0.78. It can be seen that the complexity of the system is greatly improved due to the introduction of the memristor. Such high complexity is rarely reported in related literature. In the memristor-capacitor-based chaotic system proposed by Ning Wang et al., the SE is about 0.82 with R varying [54]. And in Fractional-Order 4D Hyperchaotic Memristive designed by Jun Mou et al., the SE value is about 0.6 [55]. In other chaotic systems, SE is in the range of 0.5–0.8 [56,57,58,59,60,61,62,63,64,65,66,67]. 

Therefore, the high complexity of hiding the chaos using the memristor can provide a safer key for communication, which is of great theoretical significance for the development of chaotic security technology.

### 3.2. Entropy Analysis of Chaotic Behavior

**Figure 24:**
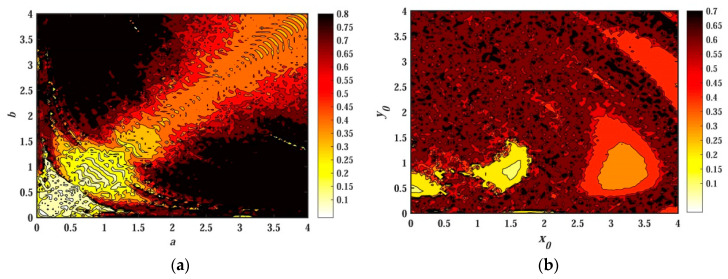
SE distribution of the system under different conditions: (**a**) the interaction of parameters a and b; (**b**) the interaction of initial values $x_{0}$ and $y_{0}$.

The nonlinear characteristics of memristor-based chaotic systems are related to many parameters. In the previous discussion, the characteristics of memristive chaotic systems are all for the case of single parameter change. In this subsection, the characteristics of chaotic systems are analyzed with the participation of two parameter variables at the same time. From the previous analysis, it can be seen that the change of system parameters and initial values is an important factor affecting the dynamic behavior of the system, which in turn affects the chaotic and SE distribution of the system. Therefore, it is of great significance to study the SE distribution of a chaotic system under the interaction of system parameters or initial values. Here, the system parameters c=e=g=1 and d=0.1 remain unchanged. The chaotic SE distribution of the system under the interaction of parameters a and b, and the change of initial value $\left(x_{0},y_{0},0,0,0\right)$ is shown in Figure 24. Figure 24a is a chaotic characteristic distribution when a∈(0,4), b∈(0,4); the initial value is (−1,−1,0,0,1), and Figure 24b is a chaotic characteristic SE distribution when $x_{0}\in \left(0,4\right)$, $y_{0}\in \left(0,4\right)$, a=1, and b=0.05.

Figure 24a reflects the SE distribution of the chaotic system under the interaction of parameters a and b. Except for a small part of the area having relatively light color distribution on the diagonal line, most of the other parts are red, dark red and black. This shows that the system is in a state of chaos within a large area and has a high degree of complexity under the combined action of parameters. Fixing the value of parameter a or b and looking along the direction of another variable, we can find that the color in the graph is fragmented. And the color in each interval is continuous, indicating that the dynamic behavior of the system is different when a parameter changes, which is highly consistent with the bifurcation diagrams.

The color distribution of Figure 24b is more dispersive than that of Figure 24a. In addition to three small areas with lighter colors, the whole area is mainly red and dark red, and the entire red area is mixed with a lot of small black spots. The black distribution color is scattered. This indicates that even when small initial values $x_{0}$ and $y_{0}$ change, there is a mutual transition between chaos and non-chaos, which indicates that the system is extremely sensitive to the initial value, and the system has multi-stability. Moreover, the diversity of colors in the figure also proves that the system has numerous hidden attractors.

In summary, Figure 24 quantitatively analyzes the nonlinear dynamic characteristics of memristor-based hidden chaotic systems from the point of view of chaotic SE distribution. The actual system parameters are much more complex than what we discussed. Therefore, the non-linear dynamic characteristics of the system are extremely rich, and there are numerous hidden attractors in the system.

## 4. Circuitry Realization of Memristor-Based Chaotic System

The dynamic characteristics and complexity of the memristor system are studied in the previous sections. This section will verify the achievability of the system by studying the design of the chaotic circuit. As we all know, achievable circuits in the system are the guarantee for applying theory to engineering applications. It means that in the nonlinear chaotic system, chaotic circuit is the core of chaos widely used in information science, and it is the technical basis for applying chaos to engineering fields such as secure communication and synchronous control. From this point of view, we will focus on the implementation of chaotic circuits based on memristors in this section. There are three methods to realize a chaotic circuit, including an individualized scheme, modular scheme and improved modular scheme [20]. Compared with the modular scheme, the individualized scheme requires less circuitry components but requires some prior experiences. The improved modular scheme can determine the circuit parameters through mathematical models. This section will use an improved modular scheme to implement the chaotic circuit.

### 4.1. Equivalent Circuit implementation for Memristor

Because the fabrication price of memristors is too high to be widely used in commerce at present, it is very attractive to employ off-the-shelf components to equal the memristor model. That is to say, the first step is to realize the mathematical model (1) using common circuit elements. After that, we design the chaotic circuit corresponding to the state Equation (3) by using the memristor model.

Observing the state of the variable phase diagrams or time domain graphs of the system, we can find that the state variables w and u are beyond the linear range of the integrated amplifiers. Therefore, the variable compression processing is required using Equation (8). After compression, system (3) becomes Equation (9).

(8) $$\left\{ \begin{array}{l} x=u_{x}\\ y=u_{y}\\ z=u_{z}\\ w=10u_{w}\\ u=10u_{u} \end{array}\right.$$

(9) $$\left\{ \begin{array}{l} \frac{du_{x}}{dt}=au_{y}+u_{x}u_{z}\\ \frac{du_{y}}{dt}=-bu_{x}+u_{y}u_{z}\\ \frac{du_{z}}{dt}=1-u_{x}{}^{2}-u_{y}{}^{2}\\ \frac{du_{w}}{dt}=-W(10u_{u})u_{w}+u_{z}u_{w}+0.1e\\ \frac{du_{u}}{dt}=u_{w}-u_{u} \end{array}\right.$$

In order to match the circuit parameters, a time constant of $\tau_{0}$ is introduced, and $\tau_{0}=RC$. Then the relationship of τ and t is $t=\frac{1}{\tau_{0}}\tau$, so there is $d\tau =\tau_{0}dt=RCdt$. Therefore, the corresponding circuit equation is shown as (10), where $g_{1}$, $g_{2}$, $g_{3}$, $g_{4}$, and $g_{6}$ are multiplier gains, and $R_{a}$ and $R_{b}$ are the branch resistors of $u_{y}$ and $u_{x}$, respectively.

(10) $$\left\{ \begin{array}{l} RC\frac{du_{x}}{d\tau }=\frac{R}{R_{a}}u_{y}+g_{1}u_{x}u_{z}\\ RC\frac{du_{y}}{d\tau }=-\frac{R}{R_{b}}u_{x}+g_{2}u_{y}u_{z}\\ RC\frac{du_{z}}{d\tau }=1-g_{3}u_{x}{}^{2}-g_{4}u_{y}{}^{2}\\ RC\frac{du_{w}}{d\tau }=-W(10u_{u})u_{w}+g_{6}u_{z}u_{w}+0.1e\\ RC\frac{du_{u}}{d\tau }=u_{w}-u_{u} \end{array}\right.$$

According to the circuit Equation (10), the memristor equation in the memristive chaotic circuit can be obtained as

(11) $$\left\{ \begin{array}{l} i=W(10u_{u})u_{w}=\left(c+10d\left|u_{u}\right|\right)u_{w}\\ \frac{du_{u}}{dt}=u_{w}-u_{u} \end{array}\right.$$

**Figure 25:**
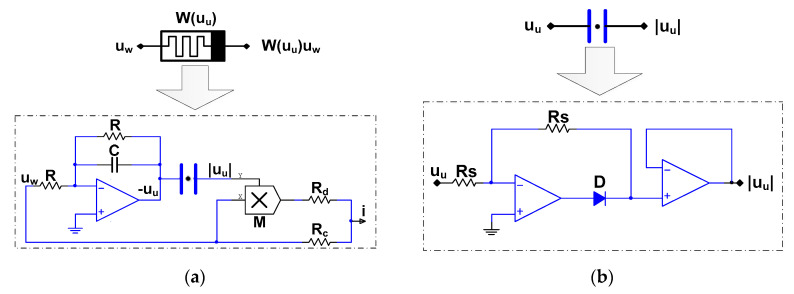
The memristor model circuit: (**a**) memristor unit; (**b**) absolute circuit.

The memristor unit circuit Equation (12) is designed by the memristor Equation (11), that is, $\frac{R}{R_{c}}=c=1$, $\frac{Rg_{5}}{R_{d}}=10d=1$, where $g_{5}$ is the multiplier gain. If R=10kΩ, $g_{5}=1$, then $R_{c}=R_{d}=10k\Omega$. Therefore, the memristor model circuit designed with the help of the basic theory of the circuit is shown in Figure 25, where the absolute equivalent circuit is shown in Figure 25b. As can be seen from the schematic diagram, we use the operational amplifier, analog multiplier, and other components to realize the analog circuit of the non-ideal flux-controlled memristor unit. The absolute circuit consists of two operational amplifiers, a diode and two linear resistors. $R_{S}=200k\Omega$ was adopted in our design.

(12) $$\left\{ \begin{array}{l} i=R\left(\frac{1}{R_{c}}+\frac{g_{5}}{R_{d}}\left|u_{u}\right|\right)u_{w}\\ \frac{du_{u}}{dt}=\frac{1}{RC}\left(u_{w}-u_{u}\right) \end{array}\right.$$

### 4.2. Circuit of Memristive Chaotic System

**Figure 26:**
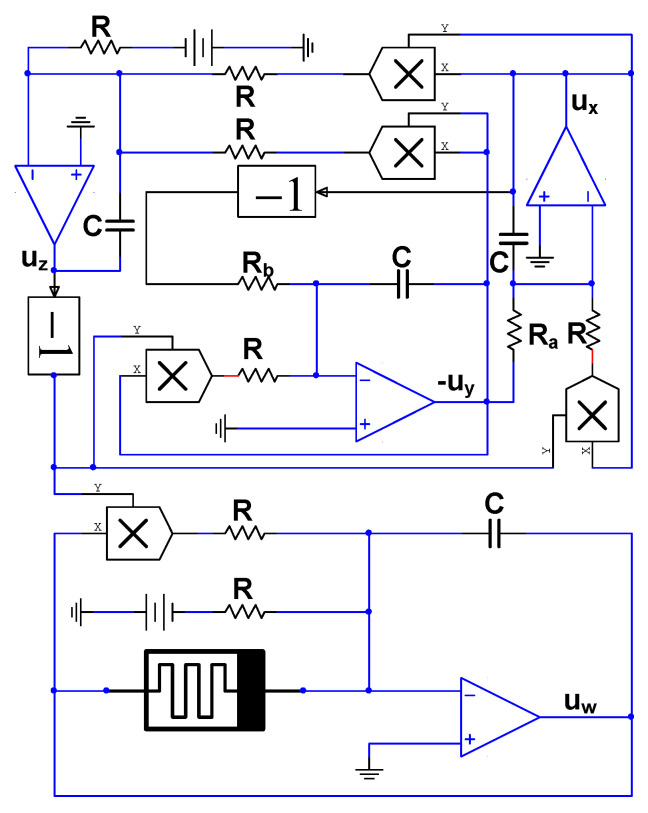
Schematic of the memristor-based chaotic system.

The previous subsection has solved the realization problem of the memristor model circuit. Based on the memristor model, we will design a chaotic system circuit containing a memristor in this subsection. By applying Kirchhoff’s law, the nature of the integrated operational amplifier, and the constraint relationship between the voltage and current of the capacitor, the circuit of the memristive chaotic system can be built in Figure 26. Here we take R=10kΩ, C=33nF. Because $\frac{R}{R_{a}}=a=1$, and $\frac{R}{R_{b}}=b=0.05$, $R_{a}=10k\Omega$, $R_{b}=200k\Omega$. The gain of all multipliers in the circuit diagram is 1, i.e., $g_{1}=g_{2}=g_{3}=g_{4}=g_{5}=g_{6}=1$.

### 4.3. Circuit simulation of Memristive Chaotic System 

**Figure 27:**
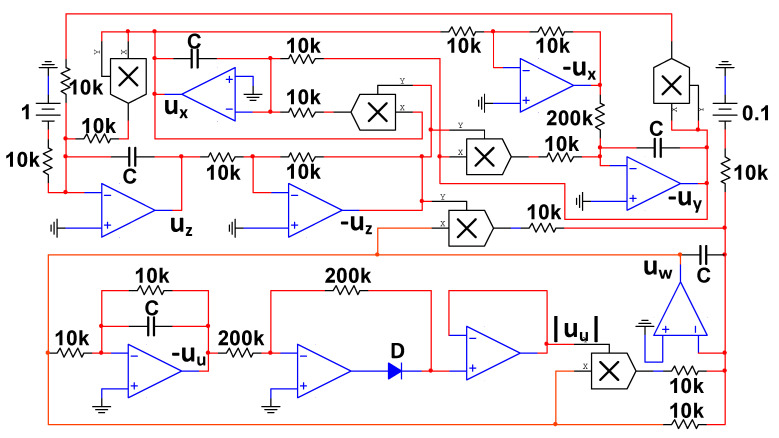
Multisim circuit simulation of system (2).

As we all know, the performance of Multisim software is very close to that of the actual component; the circuit performance of the circuit built by Multisim can approximately represent the actual circuit. Multisim has an obvious advantage over the PSIM software. According to the schematic of the five-dimensional chaotic system shown in Figure 26, the simulation circuit using the components constructed in the Multisim software is given in Figure 27. The circuit is mainly composed of operational amplifier TL082, multiplier AD633, diode 1N4148, a linear resistor, and a capacitor. In Figure 27, the power supply voltage of the operational amplifier is ±15V; the gain of all multipliers is 1; and $u_{x}$, $u_{y}$, $u_{z}$, $u_{w}$, and $u_{u}$ are consistent with the variables in the circuit Equation (9).

**Figure 28:**
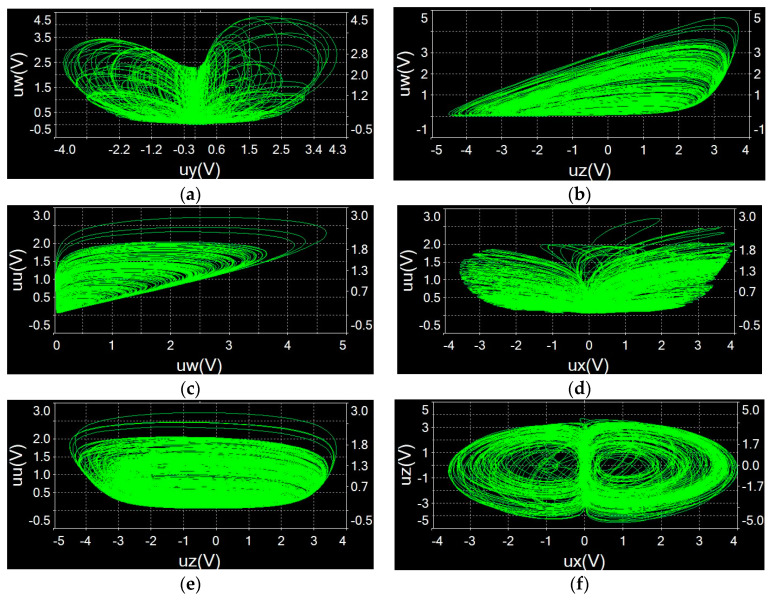
Phase diagrams observed by oscilloscope: (**a**) $u_{y}-u_{w}$ plane; (**b**) $u_{z}-u_{w}$ plane; (**c**) $u_{w}-u_{u}$ plane; (**d**) $u_{x}-u_{u}$ plane; (**e**) $u_{z}-u_{u}$ plane; (**f**) $u_{x}-u_{z}$ plane.

The Multisim simulation result observed by the oscilloscope for the chaotic system is presented in Figure 28. These results are in good agreement with numerical simulations calculated with Matlab, indicating that the hidden chaotic system composed of memristor is physically achievable.

## 5. Conclusions

In this paper, a non-ideal flux-controlled memristor model and its circuit are introduced into the four-dimensional chaotic system, and a novel and unbalanced five-dimensional chaotic system without an equilibrium point is realized. 

By analyzing the nonlinear behavior in terms of the equilibrium point, phase diagram, power spectral density map, time domain graph, LEs graph, and poincaré map, it is proved that the system has hidden chaotic characteristics. In the process of analysis, it is surprising to find that the Poincar map of the system presents a unique and complex structure different from that reported in other literature. Then, by focusing on the bifurcation diagram and LEs under different parameters of the system, it is found that the system has multiple quasi-periodic limit cycles and chaotic attractors if a certain parameter is changed. These attractors have never appeared in previous volumes and belong to new hidden attractors. After that, by studying the influence of parameter on system complexity, it is found that the value of SE can reach about 0.87, which is very rare in other chaotic systems. At the same time, the chaotic behavior of SE is analyzed by using the contour line method. It is found that the memristor-based hidden chaotic system has a very sensitive initial value, multi-stability and innumerable hidden attractors. That is to say, under different initial conditions, the memristive system exhibits the coexistence of chaotic attractors with different topological structures, quasi-periodic limit cycles, chaotic attractors, and the multi-attractor phenomena of quasi-periodic limit cycles and chaotic attractors with different topological structures. These nonlinear characteristics prove that the system has a very high complexity after introducing memristors.

In addition, we also find novel and interesting quasi-periodic and chaotic attractors that are different from other attractors in transient chaos. Finally, the consistency of theoretical analysis and numerical simulation results is verified by the design of an analog circuit. The physical realization of the memristor-based hidden chaotic system provides a possibility for its application in engineering technology.
